# Optimization of CRISPR/Cas9 genome editing to modify abiotic stress responses in plants

**DOI:** 10.1038/srep26685

**Published:** 2016-05-26

**Authors:** Yuriko Osakabe, Takahito Watanabe, Shigeo S Sugano, Risa Ueta, Ryosuke Ishihara, Kazuo Shinozaki, Keishi Osakabe

**Affiliations:** 1 RIKEN Center for Sustainable Resource Science, 3-1-1 Koyadai, Tsukuba, Ibaraki 305-0074, Japan; 2 Center for Collaboration among Agriculture, Industry and Commerce, Tokushima University, 3-18-15 Kuramoto-cho, Tokushima 770-8503, Japan; 3 Faculty of Bioscience and Bioindustry, Tokushima University, 2-1 Josanjima, Tokushima 770-8513, Japan

## Abstract

Genome editing using the CRISPR/Cas9 system can be used to modify plant genomes, however, improvements in specificity and applicability are still needed in order for the editing technique to be useful in various plant species. Here, using genome editing mediated by a truncated gRNA (tru-gRNA)/Cas9 combination, we generated new alleles for *OST2*, a proton pump in Arabidopsis, with no off-target effects. By following expression of Cas9 and the tru-gRNAs, newly generated mutations in CRIPSR/Cas9 transgenic plants were detected with high average mutation rates of up to 32.8% and no off-target effects using constitutive promoter. Reducing nuclear localization signals in Cas9 decreased the mutation rate. In contrast, tru-gRNA Cas9 cassettes driven by meristematic- and reproductive-tissue-specific promoters increased the heritable mutation rate in Arabidopsis, showing that high expression in the germ line can produce bi-allelic mutations. Finally, the new mutant alleles obtained for *OST2* exhibited altered stomatal closing in response to environmental conditions. These results suggest further applications in molecular breeding to improve plant function using optimized plant CRISPR/Cas9 systems.

Targeted genome editing using clustered regularly interspaced short palindromic repeats (CRISPR) and CRISPR-associated protein9 nuclease (Cas9) is one of the most effective genome editing technologies developed in recent years, and is utilized widely for genetic engineering in various organisms including plants^1^,^2^,^3^,^4^,^5^,^6^,^7^,^8^,^9^. In the CRISPR/Cas9 system, Cas9 endonuclease, an RNA-guided nuclease from *Streptococcus pyogenes*, can be engineered to target specific DNA sequences using recognition via complementary base pairing^1^,^6^. In the target DNA sequence for RNA-guided Cas9 nuclease, 5′-N20-NGG-3′, NGG, the protospacer-adjacent motif (PAM) plays an important role in the formation of the Cas9/gRNA/DNA complex. CRISPR/Cas9 system has been developed in model plants such as *Arabidopsis*^5^,^10^,^11^,^12^ and tobacco^5^,^7^,^12^, and is now utilized effectively in rice with high efficiency^10^,^11^,^12^,^13^, and also in other crop species, such as sorghum^12^, wheat^14^, maize^15^, sweet orange^16^, and soybean^17^,^18^, and woody plants such as poplar^19^,^20^. The CRISPR/Cas9 system has also been used effectively in the liverwort *Malcantia polymorpha*, which has a haploid-dominant life cycle, allowing the numerous advantages to be applied to molecular genetic studies in a model species for basal land plants^8^.

Non-specific cleavages, causing so-called “off-target” mutations, can occur during genome editing and represent a challenge that must be overcome, especially for clinical applications and in crop breeding. Several strategies aimed at eliminating off-target effects and improving the specificity of CRISPR/Cas9 have been reported^21^,^22^. The combination of a mutated version of Cas9 (D10A, Cas9 nickase; Cas9n) and carefully designed gRNAs can improve the specificity of CRISPR/Cas9^1^,^3^,^21^,^23^ and are effective in avoiding off-target effects^24^,^25^. gRNAs truncated at the 5′ end (17–18 nucleotides) can also decrease off-target mutations^22^. In addition, it has been reported that optimization of gRNA and Cas9 expression levels can increase specificity^21^,^22^,^25^. In plant genome editing, off-target efficiency has been evaluated using Cas9 nickases^26^,^27^, whereby off-targets might be avoided by using two adjacent sgRNA target sequences to guide the Cas9 nickases to generate a DNA double-strand break (DSB) at the target locus. However, this remains an area where further studies are still needed to eliminate off-target effects in plant genome editing.

Here, we report an efficient system using truncated-gRNAs (tru-gRNAs) in the CRISPR/Cas9 system to produce site-directed modifications in plants with no off-target effects. We focused on mutation of an abiotic stress-responsive factor in order to evaluate modification of a functional gene in plants, specifically the gene encoding OPEN STOMATA 2 (OST2) (AHA1)—a major plasma membrane H^+^-ATPase in stomata response in Arabidopsis^28^.

Plasma membrane proton (H^+^)-ATPases play an important role in the generation of proton gradients in plant cells, activating various secondary transporters including the uptake of ions and metabolites^29^,^30^. There are eleven members of the plasma membrane H^+^-ATPases in Arabidopsis, AHA1–AHA11^31^, which are composed of N-terminal and C-terminal domains in the cytoplasm, and a transmembrane domain consisting of 10 helices including phosphorylation and nucleotide-binding sites^32^. The C-terminus is the major regulatory domain involved in inhibition of H^+^-ATPase, and activation is controlled by phosphorylation in this region and subsequent interaction with 14-3-3 proteins^33^. Two dominant mutations in the *ost2* locus abolish stomata responses to abscisic acid (ABA), leading to constitutive activity of the proton pump^28^.

We applied our improved CRISPR/Cas9 system to introduce novel alleles of *OST2* in Arabidopsis using co-expression of Cas9 and GFP via the 2A-coupled system to monitor and select Cas9-expressing cells^34^. The expression levels of Cas9, GFP and tru-gRNA were monitored and evaluated in transgenic plants, and the use of a tru-gRNA as a means of eliminating off-target effects was evaluated. The heritable efficiency in subsequent generations was increased using a tru-gRNA guided Cas9 driven by a promoter with high expression specifically in the germ line. The novel alleles for *OST2* exibited altered stress responses in Arabidopsis. These results suggest further applications of CRISPR/Cas9-mediated genome editing to improve plant growth and stress responses.

## Results

**Figure 1:**
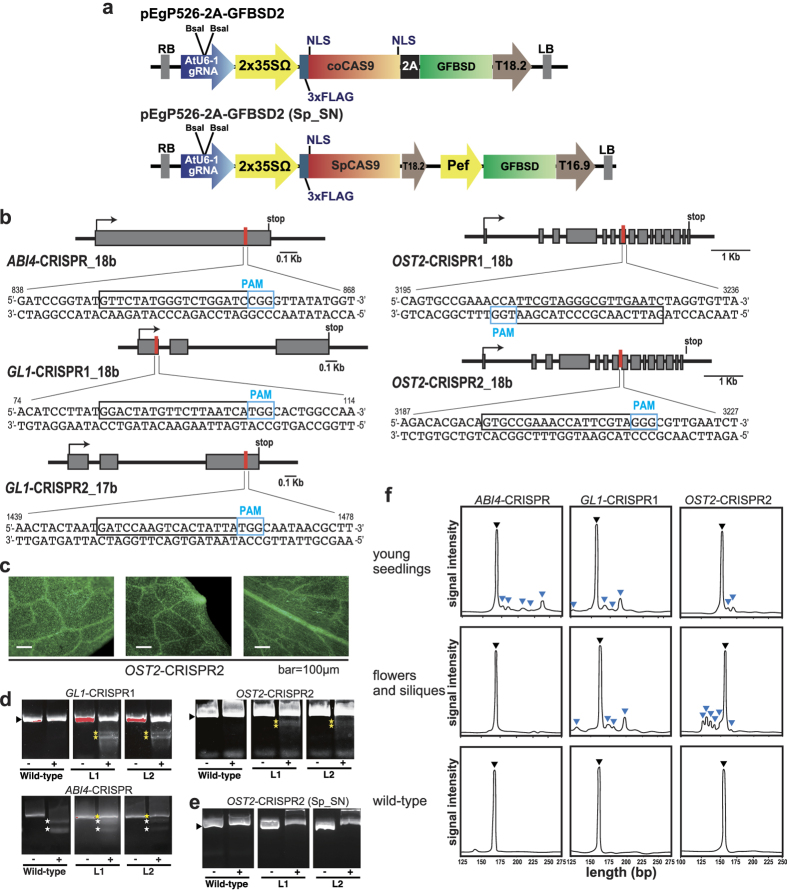
Schematic illustrating the CRISPR/Cas9 vectors using truncated gRNAs, target sites in the Arabidopsis genes, and mutation detection. (**a**) Schematic structure of the CRISPR/Cas9 vectors used in this study. The plant codon-optimized Cas9 with 3 × FLAG and 2 × NLSs was inserted in front of the 2A peptide fused with GBBSD2 under the control of the 2 × CaMV 35S promoter. The tru-gRNA can be inserted into the *Bsa* I site and the *AtU6-1* promoter was used in the expression cassette. Cas9 with a single NLS was also used to assess the effect of NLS number on mutation efficiency. The vectors were named pEgP526-2A-GFBSD2 and pEgP526-2A-GFBSD2 (Sp_SN), respectively. (**b**) Target sequences for mutagenesis with tru-RNA-guided genome editing in the Arabidopsis genome. The selected target sequences (17–18 b) for each gene are shown in black boxes and the PAM sequences are light blue boxes. Arrows; translational start sites (+1), Gray boxes; exons. (**c**) GFP fluorescence in the T1 transgenic plants of the *OST2*-CRISPR2 (pEgP526-2A-GFBSD2). Bar = 100 μm. (**d**) Cel-1 analysis of T1 transgenic plants of *GL1*-CRISPR1 and *OST2*-CRISPR2 (pEgP526-2A-GFBSD2) and PCR-RFLP analysis of *ABI4*-CRISPR (pEgP526-2A-GFBSD2) with high GFP fluorescence compared with the wild-type control. Black arrows indicate the PCR products for wild-type sequence and yellow stars show the mutated bands digested with Cel-1 nuclease (*GL1*-CRISPR1 and *OST2*-CRISPR2) or undigested in PCR-RFLP (*ABI4*-CRISPR). L1, L2; individual T1 lines for transgenic plants. (**e**) Cel-1 analysis of T1 plants of *OST2*-CRISPR2 transformed with the single NLS-Cas9 cassette {pEgP526-2A-GFBSD2 (Sp_SN)}. (**f**) Heteroduplex mobility assay (HMA) T1 plants transformed with the CRISPR/Cas9. Multiple heteroduplex peaks (blue triangles) were detected in PCR amplicons from each of the CRISPR/Cas9-transformed plants, whereas a single peak was detected from each wild-type control (black triangle).

We engineered plant CRISPR/Cas9 vectors in which the following components exist in the same all-in-one vector: the fungal and plant codon-optimized Cas9 (fcoCas9) fused to *GFBSD2* (GFP and the brastcidin resistance fusion gene^35^) via peptide 2A, driven by the 35S CaMV promoter, and a tru-gRNA expression cassette under control of the *AtU6-1* promoter (Fig. 1a). With this vector, GFP expression can be used to monitor and select Cas9-expressing cells. Multiple nuclear localization signal (NLS) sequences control nuclear import tightly, and Cas9 with NLS sequences at both N- and C-termini has been shown to ensure efficient nuclear targeting^5^,^10^ (Fig. 1a). To test the effect of the number of NLS sequences on basal Cas9 activity in plant cells, we also constructed an SpCas9 cassette with a single NLS at the N-terminus (Fig. 1a).

Tru-gRNAs that are truncated at the 5′ end (17–18 nucleotides) have been shown to decrease off-target effects in mammals^22^. To evaluate mutation and off-target rates when using tru-gRNAs in plant cells, we designed 17–18 bp tru-gRNAs targeted towards several genes functioning in stress response and plant growth in Arabidopsis: *ABSCISIC ACID INSENSITIVE4* (*ABI4*)^36^, *GLABRA1* (*GL1*)^37^, and *OST2*^28^ (Fig. 1b). Each of these tru-gRNAs was subjected to in-silico analysis using the new gRNA design website “focas” (http://focas.ayanel.com/), which we set up as a user-friendly interface to find potential off-target sites using the CasOT algorithm^38^. Using this tool, we can also evaluate on-target activity *in silico* using the algorithm “on_target_score_calculator.py”^39^ and thus obtain an *in silico* recommended ranking of gRNAs (see Materials and Methods). The selected tru-gRNAs for each gene were cloned into the CRISPR/Cas9 vector described above, and the constructs were named *ABI4-CRISPR*, *GL1-CRISPR1*, *GL1-CRISPR2*, *OST2-CRISPR1*, and *OST2-CRISPR2* (Fig. 1b).

To evaluate mutation rates using these CRISPR/Cas9 cassettes, we selected transgenic T1 Arabidopsis plants by detecting GFP fluorescence in leaves (Fig. 1c). The selected T1 transgenic plants showed strong GFP fluorescence, which should also indicate good coexpression of Cas9 (Fig. 1c). Next, a Cel-1 assay or PCR-RFLP analysis was carried out using genomic DNA from CRISPR/Cas9 transgenic plants of *GL1-CRISPR1* and *OST2-CRISPR2* or *ABI4-CRISPR*. Digested bands in Cel-1 assay due to a heteroduplex of mutant and wild-type DNAs were detected in the PCR amplicons from almost all GFP-positive T1 individuals in *GL1-CRISPR1* (100%; 12 Cel-1 positives in 12 GFP positives) and *OST2-CRISPR2* (93%; 14 Cel-1 positives in 15 GFP positives) derived from CRISPR/Cas9 cassettes using Cas9 with double NLSs (Fig. 1d). PCR-RFLP bands that remained undigested due to a mutation were detected in PCR amplicons from all GFP-positive T1 individuals for *ABI4-CRISPR* (100%; 13 PCR-RFLP positives in 13 GFP positives) (Fig. 1d). In contrast, no digested bands were detected in the Cel-1 assay of *OST2-CRISPR2* when Cas9 with a single NLS was used (Fig. 1e), suggesting the importance of sufficient nuclear signaling of Cas9 in mediating CRISPR/Cas9 site-directed mutagenesis in plant cells. In the Cel-1 assay for *GL1-CRISPR2* and *OST2*-CRISPR1, no digested bands were detected, suggesting quite a low mutation efficiency (data not shown).

**Figure 2:**
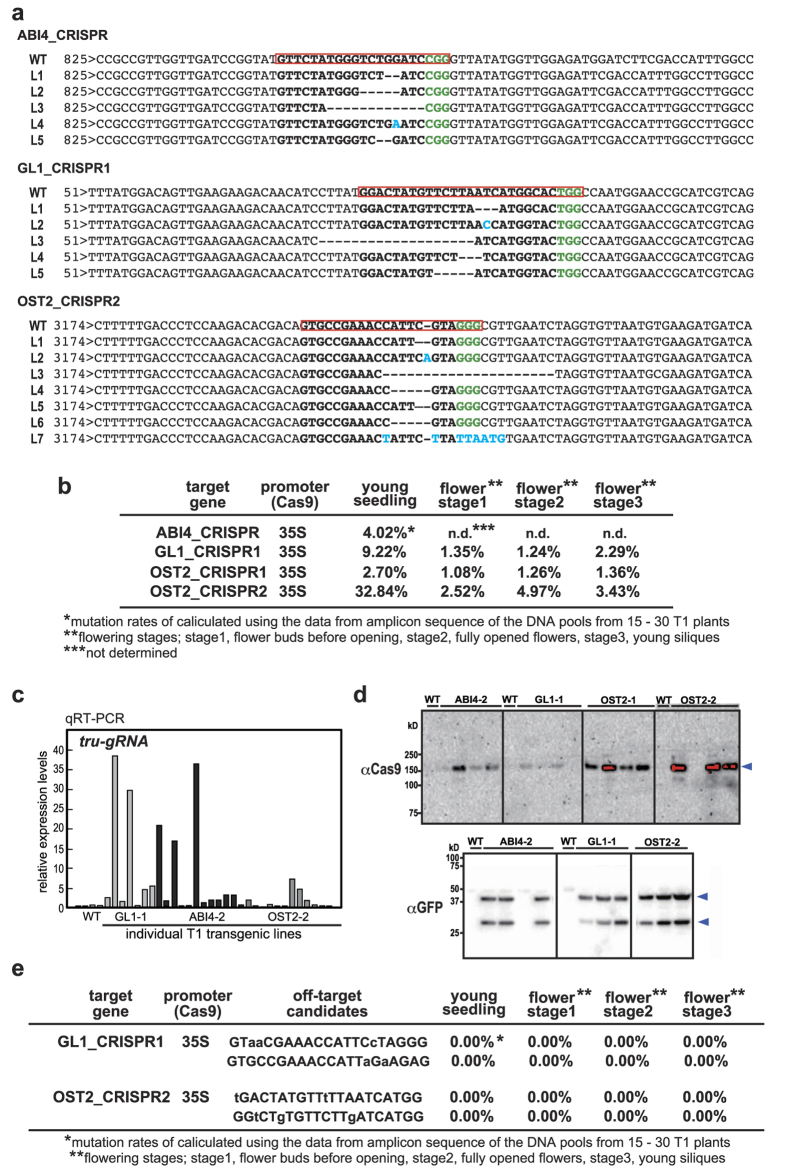
Analysis of the mutated sequences induced by tru-gRNAs. (**a**) Various types of CRISPR/Cas9-induced mutation detected by amplicon sequencing in T1 plants. The red boxes indicate target sites and green characters are PAM sequences. The blue characters show the nucleotide insertion. L1-7; the individual T1 lines for the transgenic plants. (**b**) The mutation rates analyzed by next-generation amplicon-based deep sequencing using mixed DNA pools from 15–30 GFP-positive T1 plants. (**c**) The detection of *sgRNA* expression levels in individual T1 Arabidopsis plants with each *sgRNA* specific primer with qRT-PCR. The value for one of the T1 lines of each construct was set to 1.0. To normalize the expression levels, 18S rRNA was amplified as an internal control. WT; wild-type Arabidopsis. (**d**) Protein blot analysis of GFP and Cas9 was performed with the individual T1 plants using an anti-GFP and Cas9 antibody, respectively. (**e**) The off-target mutation rates analyzed by next-generation amplicon-based deep sequencing using mixed DNA pools from 15–30 GFP-positive T1 plants. The off-target rates were shown by calculating the differences between these values and those from wild-type Arabidopsis (data not shown).

To investigate mutation tendency at different plant stages, we next performed a heteroduplex mobility assay (HMA) using microchip electrophoresis in PCR amplicons from different plant stages in GFP-positive T1 plants (Fig. 1f). Multiple heteroduplex peaks were observed in PCR amplicons of the GFP-positive *ABI4-CRISPR*, *GL1-CRISPR1*, and *OST2-CRISPR2*, whereas a single peak was detected in the wild-type (Fig. 1f), *GL1-CRISPR2*, and *OST2*-*CRISPR1* (data not shown), indicating that CRISPR/Cas9-mediated indels were induced at the target locus in *ABI4-CRISPR*, *GL1-CRISPR1*, and *OST2-CRISPR2*. Furthermore, the variation and pattern of mutations during plant development differed for each of the CRISPR/Cas9 constructs (Fig. 1f). We analyzed the sequences of sub-cloned PCR products from individual T1 plants that had high mutation rates detected by Cel-1 (Fig. 2a). Using next-generation amplicon-based deep sequencing, we then analyzed the sequences of mixed DNA pools at several developmental stages from 15–30 GFP-positive T1 plants (Fig. 2b and Supplemental Fig. 1). The results showed that *ABI4-CRISPR*, *GL1-CRISPR1*, and *OST2-CRISPR2* could introduce DSB and induce indels at the target genomic sites. Analysis of the mutant sequences indicated that the variety of mutation types and average mutation rates in all targets were higher in DNA pools from young seedlings than in those from flowering stages (Fig. 2a,b and Supplemental Fig. 1). In particular, nucleotide insertions in the 3- or 4-bp upstream of the PAM sequence were most commonly observed in DNA pools of the T1 Arabidopsis plants (Supplemental Fig. 1). The highest rate of 32.8% (mutated reads/total reads) was obtained in *OST2-CRISPR2* plants (Fig. 2b), and the data suggest that the mutation rate varied depending on the tru-gRNA target sequence.

The mutations in the T1 plants were dependent on each tru-gRNA, *OST2-CRISPR1* and *OST2-CRISPR2*, with closely spaced target sequences at the *AHA1/OST2* locus, exhibiting very different mutation efficiencies. To investigate if mutation efficiency correlates with Cas9 and tru-gRNA expression, the expression levels of *Cas9* and *tru-gRNAs* in GFP-positive individuals were monitored using quantitative RT-PCR (Fig. 2c and Supplemental Fig. 2). We also analyzed GFP levels to monitor the 2A-coupled Cas9/GFP system. The expression levels of *Cas9* and *GFP* (driven by the *35SCaMV* promoter), and *tru-gRNA* (driven by *AtU6-1* promoter) varied in individual T1 plants, suggesting that the locus into which the T-DNA had inserted, and the copy number, were the factors that most affected expression levels in each individual. GFP and Cas9 protein levels were also evaluated by western blot (Fig. 2d); two bands for GFP, which might correspond to full-length GFBSD2 and truncated GFP, were detected (Fig. 2d).

We next evaluated the off-target effects of tru-gRNAs with higher mutation efficiency, *OST2-CRISPR2* and *GL1-CRISPR1* (32.84% and 9.22%, respectively), in Arabidopsis. In the CasOT off-target analysis of *OST2-CRISPR2* gRNA, the most likely off-targets were 5′-GTaaCGAAACCATTCcTAGGG-3′ (locus; *At3g55650*) and 5′-GTGCCGAAACCATTaGaAGAG-3′ (locus; *At5g57350*) (3 b and 2 b mismatches in lower case), and *GL1-CRISPR1* off-targets were 5′-tGACTATGTTtTTAATCATGG-3′ (locus; *At3g59310*) and 5′-GGtCTgTGTTCTTgATCATGG-3′ (locus; *At1g15050*) (2 b and 3 b mismatches in lower case). We then used next generation amplicon sequencing to analyze these sequences in *OST2-CRISPR2* and *GL1-CRISPR1* transgenic T1 plants (Fig. 2e). Sequence analysis indicated that no off-target mutations were detected in the T1 DNA pools for each target, suggesting that the tru-gRNAs for *OST2-CRISPR2* and *GL1-CRISPR1* induced DSBs efficiently and specifically only at the specified on-target sequence, and the various mutations were then introduced via subsequent non-homologous end-joining repair.

We next analyzed *OST2-CRISPR2*-induced mutations in the T2 generation derived from the T1 line with the highest mutation rate (~60% in the T1 cloned DNA; data not shown). Cel-1 and sequence analysis of the sub-cloned PCR amplicons indicated a high efficiency of mutation in *OST2-CRISPR2* T2 plants (Supplemental Fig. 3). The results indicated that the high expression of *Cas9* nuclease in plant cells driven by the strong constitutive *CaMV35S* promoter caused a “mosaic” effect with high mutation rates, and that the low expression in the germ line^40^ would affect the rate at which mutated homozygotes were generated, especially during *in planta* transformation as previously suggested.

**Figure 3:**
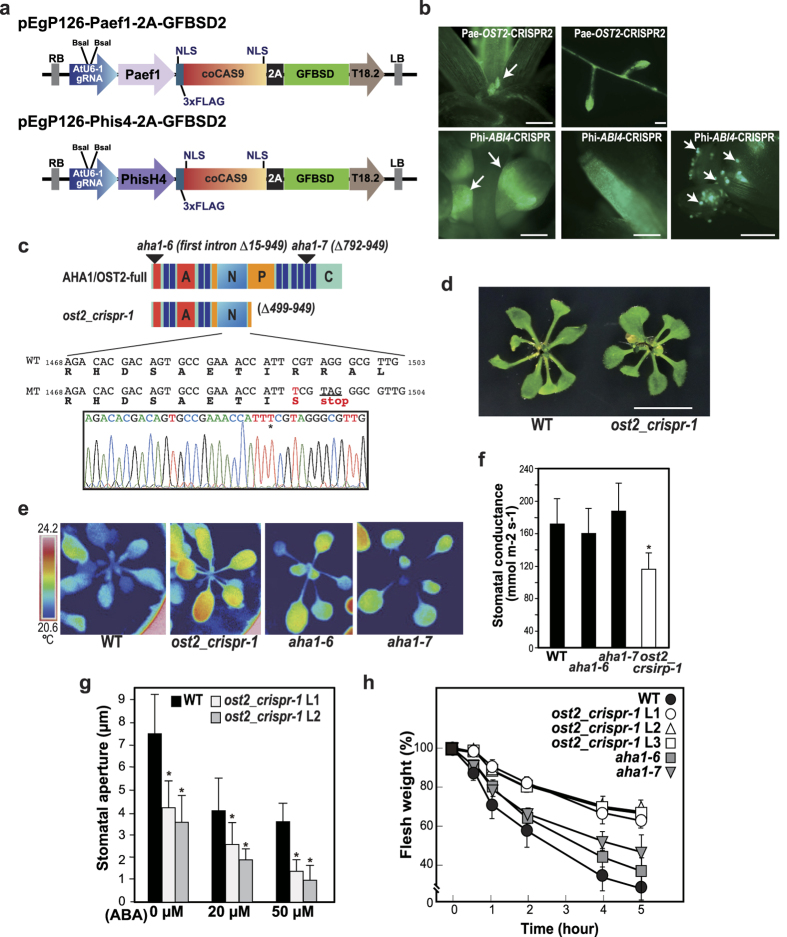
Generation of a new allele of the ost2 mutant using a tru-gRNA and Cas9 driven by a tissue-specific promoter, and the altered stomatal responses in Arabidopsis. (**a**) Schematic structure of the CRISPR/Cas9 vectors harboring a tissue-specific promoter for Cas9 expression using Arabidopsis *elongation factor α-1* (*AtEF1*) (pEgP126_Paef1-2A-GFPSD2) and *histoneH4* (*AtH4*) (pEgP126_Phis4-2A-GFPSD2) promoters. (**b**) GFP fluorescence in the T1 transgenic plants of the tissue-specific CRISPR/Cas9 vectors. Bar = 100 μm. (**c**) Generation of a new allele of *ost2* mutant (*ost2_crispr-1*) using the tissue-specific CRISPR/Cas9. A base substitution (1492C to T, red character and asterisk in the sequence data) was detected in genomic DNA in the mutants. This mutation creates a stop codon (underlined) just after the mutation site. (**d**) A new mutant for *OST2* gene (*ost2_crispr-1*) generated by the tissue-specific CRISPR/Cas9. Bar = 1 cm. (**e**) Thermal infrared imaging of the wild-type and *ost2_crispr-1* showing the temporal variability in stomatal conductance. (**f** ) Stomatal conductance of rossette leaves in the wild-type and *ost2_crispr-1* measured by leaf polometer. Values are means and SD (*n* = 20). Asterisks indicate statistically significant differences between the wild-type and mutant plants, determined by Student’s *t* tests. *P < 0.0001 (**g**) Effects of various concentrations of ABA on stomatal closure in *ost2_crispr-1* and wild-type. Epidermal peels were treated with or without ABA for 1 h after stomatal preopening under light for 2 h, and the stomatal aperture was measured under a microscope. Values are means and SD (*n* = 30). Asterisks indicate statistically significant differences between the wild-type and mutant plants, determined by Student’s *t* tests. *P < 0.0001. L1, L2 are T2 individuals harboring mutations in OST2 locus. (**h**) Transpirational water loss in *ost2_crispr-1* and wild-type at the indicated time points. Water loss is expressed as a percentage of the initial fresh weight. Values are means and SD of five samples of two shoots of each *ost2_crispr-1* and wild-type.

To create novel mutant alleles of the target gene in Arabidopsis, we then constructed new vectors, in which *Cas9* expression is controlled by strong tissue-specific promoters, *elongation factor α-1*^41^ and *histone H4* promoters^42^ (Fig. 3a). These promoters directed high GFP fluorescence in a tissue-specific manner, being expressed especially highly in shoot tips, young leaves, anthers, pollens, and root tips (Fig. 3b). High tissue-specific expression of *Cas9* in meristematic tissues and germline presumably causes low-efficiency mutation in somatic tissues of the T1 generation of Arabidopsis, and the expected result, i.e., a low mutation efficiency in *OST2-CRISPR2* T1 plants, was obtained (data not shown). A similar result has also been reported using a CRISPR/Cas9 cassette in which the *Cas9* expression was driven by the flower-specific *APETALA1* promoter^43^. The high mutation rates of the *OST2-CRISPR2* tru-gRNA were confirmed as described above; we next generated T2 plants to identify single *OST2-CRISPR2* mutant lines using tissue-specific promoter CRISPR/Cas9 cassettes with a 97% mutation rate in the 20% T2 lines (two bi-allelic lines/10 T2 lines) (Supplemental Fig. 4a). We also examined mutations in T2 individuals of *ABI4-CRISPR* driven by the tissue-specific promoter, by analyzing plant phenotypes in ABA sensitivity (Supplemental Fig. 4b). The results indicated that mutations were generated in the T2 plants with high efficiency using tissue-specific CRISRP/Cas9 cassettes.

In the isolated *OST2-CRISPR2* homozygous mutant, a 1-bp insertion at the target site caused a frameshift and introduced a stop codon at the codon (R498) neighboring the mutation site to create a dominant-negative mutation of OST2 (Fig. 3c). We then investigated if the novel mutant allele affects plant phenotype in terms of response to abiotic stress. A T3 homozygote of the mutant line of *OST2-CRISPR2* was isolated, namely *ost2_crispr-1*. An *ost2-1D* mutant that impairs guard cell responses completely has been isolated previously based on lower leaf temperature visualized by infrared imaging, and displayed a growth retardation phenotype^28^,^44^. In contrast, *ost2_crispr-1* did not show any significant differences in plant growth compared with wild-type plants (Fig. 3d). Infrared imaging of the leaves of *ost2_crispr-1* revealed that the leaf temperature in *ost2_crispr-1* mutants was clearly higher than that in wild-type plants, suggesting enhanced stomatal closure in *ost2_crispr-1* (Fig. 3e). Furthermore, stomatal conductance of *ost2_crispr-1* leaves decreased compared to that of wild-type leaves (Fig. 3f). T-DNA insertion mutants *aha1-6* and *aha1-7*^45^ were also analyzed, suggesting weak phenotypes (Fig. 3e,f). H^+^-ATPases play major roles in stomata opening in response to environmental signals such as light^46^. Infrared imaging of *ost2_crispr-1* suggested that the mutation introduced by CRISPR/Cas9 negatively affected OST2 activity in stomatal opening.

We next analyzed the stomatal responses of *ost2_crispr-1* in more detail. ABA controls turgor pressure in guard cells to induce stomatal closure, and H^+^-ATPase activity is inhibited by the ABA signaling pathway^28^. We investigated the *ost2_crispr-1* mutation in ABA-induced stomatal closure in Arabidopsis, and found the rate of stomata closing of *ost2_crispr-1* to be higher than those of wild-type (Fig. 3g). A lower rate of transpirational water loss as determined by measuring the fresh weight of detached leaves of *ost2_crispr-1* was also observed compared with that of wild-type and T-DNA insertion mutants *aha1-6* and *aha1-7*^45^ (Fig. 3h); these data indicate enhanced stomatal response in *ost2_crispr-1* plants.

## Discussion

The new approach to genome editing using the CRISPR/Cas9 system has now been applied widely to many plant species including crop plants^9^. We developed procedures to monitor and validate CRISPR/Cas9 expression, and to optimize mutation efficiency, first by using tru-gRNAs with no off-target effects in plants. Since Cas9 nuclease expression levels greatly influence mutation efficiency, we employed a 2A-coupled system to co-express Cas9 and GFP in the same plant cells to select plants with high nuclease levels. Genome editing with 2A peptide-coupled co-expression of fluorescent protein and nuclease, combined with cell sorting, has been shown to allow high genome editing rates in human cells^34^. We showed here that this method can also be used to generate mutations in the plant genome. Direct comparison of the expression levels of both RNA and protein of Cas9 and GFP in the Arabidopsis plants suggested that mutation rates were not correlated strongly with expression levels; however, the high expression levels of Cas9 were sufficient to direct effective genome editing. We also developed a detection system for sgRNA expression levels in plant cells; the results showed that sgRNA levels driven by the *AtU6-1* promoter varied greatly depending on the specific CRISPR/Cas9 introduced into each plant. These results might suggest that the fine-tuning of adequate expression levels of both tru-gRNA and Cas9 protein together might allow high mutation rates in plant cells. The several types of pol III promoter required for gRNA expression could be used to improve gRNA expression levels in plant cells. Taken together, the system for monitoring Cas9 levels, together with optimization of gRNA expression, will improve one of the useful genome editing methods for plant cells, including crops, especially using tissue culture systems that need to select cells with high expression.

In this study, we first investigated truncated version of gRNAs with target sequences shorter than 20 nucleotides in plant cells with a view to improving the specificity of CRISPR/Cas9 with low off-target effects^22^. We used 17–18b tru-gRNAs for several target sequences in genes raelated to stress responses in Arabidopsis, and found that 18b tru-gRNA especially could work effectively in plant cells. Interestingly, two types of tru-gRNA targetting almost the same genomic site in *OST2* showed different mutation rates. The on-target scores calculated using “focas” for the 20-bp version of *OST2*_CRISPR1 and *OST2*_CRISPR2 were 0.33 and 0.51, respectively, showing that a “focas” on-target score might correlate with activity. The mutation rates associated with the tru-gRNAs varied regardless of gRNA length, and seemed to be affected strongly by each tru-gRNA target sequence, as is also the case with the more usual 20-b gRNAs. Differences in the pattern of mutations have been observed in different plant species^5^. In this study, varied patterns of mutation were also detected during plant development for each tru-gRNA. These results suggest the possibility that the developmental stage of mutation, as well as the design of the gRNA, might affect biding of both gRNA and Cas9, stability of the DNA repair factors, and/or selection of the DNA repair pathway.

We evaluated off-target effects with tru-gRNAs in plant cells using the amlicon sequences of candidate off-targets, and found that the tru-gRNAs effectively generated mutations with no off-target effects in plant cells. Thus, tru-gRNAs could effectively be used in the further application of plant genome editing, especially in important crop species in which the acquirement of single mutant lines is quite difficult and propagation by tissue culture only has to be applied. Recent studies have suggested that off-target mutations using 20 bp gRNAs are not detected as frequently in plants as in mammalian system^47^. However, further studies are needed to allow a precise comparison of off-target effects between tru-RNAs and 20nt gRNAs targeted to the same locus.

Genetic transformation in most plant species, including crops, utilizes a tissue culture step, and genome modification usually occurrs during proliferation in callus cells. On the contrary, heritable mutations can generaly be evaluated in Arabidopsis, in which an *in planta* transformation system can be used for the introducation of CRISPR/Cas9^5^,^10^,^11^,^12^. In the plant CRISPR/Cas9 system, the genome modification that occurs in the early stages of development involves heterozygous, homozygous, and bi-allelic mutations, and an intermingling of these mutation types is generally observed (“mosaic” or “chimera”). A means of screening homozygous mutants from among these mutation types is needed, and, in Arabidopsis, the T2 and subsequent generations can be evaluated to obtain homozygotes. The low strength of the constitutive promoters that drive *Cas9* expression in germline and egg-cells are presumably the cause of the highly mosaic plants and low levels of homozygote plants. To improve these issues, we employed strong tissue-specific promoters in the meristematic tissues and germline to control *Cas9* expression in Arabidopsis. Our results showed a significant improvement in heritability when comparing use of the 35S promoter and the same tru-gRNA in Arabidopsis, and subsequent generations can be used for analysis of phenotype. Recently, to obtain homozygous mutants in early segregation in Arabidopsis, several approaches applying tissue-specific promoters have also been evaluated: the *INCURVATA2* (*ICU2*) promoter that has an S-phase specific *cis*-element for expression in the meristematic region and embryo^48^, the *APETALA 1* (*AP1*) promoter for expression in floral meristematic regions^43^, the *DD45/EC1.2* promoter with egg cell-specificity to expect expression in the one-cell stage embryo^47^, the *YAO* promoter in tissues undergoing active cell division^49^, and the *SPOROCYTELESS* (*SPL*) promoter with germ line-specificity^50^. Wang *et al.* also used double gRNA expression cassettes in their system, resulting in efficient mutation^47^. These approaches represent strong tools for heritable mutation in plants, especially via genome editing using *in planta* transformation.

Using the tissue-specific Cas9 cassette guided by a tru-gRNA, we obtained a new mutant allele for *OST2* (*ost2_crispr-1*) in Arabidopsis, and found that it increased stomatal responses. Based on the crystal structure of AHA2, an OST2 homologous protein, the H^+^-ATPase is generally composed of an A (actuator)-domain, M (membrane)-domains, P (phosphorylation)-domain, N (nucleotide binding)-domain , and R (regulatory)-domain (Fig. 3c)^32^,^51^. *ost2_crispr-1*mutation generated a stop codon just after the P-domain and deleted the N, M (M5-M10), and R-domains. Disruption of the M domains that form the intramembrane cavity and contain amino acids important for proton binding, and the N domain, which is essential for activity, would strongly repress OST2 activity. It has been suggested that plant H^+^-ATPase forms a dimeric protein complex, and that dimerization is related to the activation mechanism^51^. The truncated version of OST2 might affect dimerization of other AHA proteins with redundant function and repress their activity. Our results, in which the OST2 mutant exhibited clear phenotypes in its stomatal responses, might support this hypothesis. Genetic studies showing that an *aha1* and *aha2* double knockout is embryo-lethal, whereas a single knockout does not show any strong phenotype, and the *AHA1* or *AHA2* transgene rescues the lethality, also suggest an overlapping function of these proteins^45^. The dominant mutations would have a greater effect than the simple knockout mutations, and the *ost2_crispr-1* mutation indicated that this is indeed the case, suggesting further applications showing how mutations can be generated in target genes by genome editing.

In conclusion, we have developed an effective genome editing system based on Cas9 and a tru-gRNA complex that can effectively generate mutations without off-target effects in plant genomes. This method would offer addvantages in various crop species, especially those whose draft genomes have only recently been made available. We also found tissue-specific expression of Cas9, in which the levels of Cas9 with adequate NLSs were associated with GFP expression and truncated gRNA in plant cells. The newly generated mutant for the stress-related gene *OST2* increased stomatal response. Our results suggest that these improvements to the CRISPR/Cas9 system will make an important contribution to genome editing in plants. Moreover, it will also be important to develop a system to evaluate specific gRNAs that have high mutant efficiency, especially to generate GT systems in various plant species. To design novel mutations for target genome sites using CRISRP/Cas9, we can use existing information on protein domain structures, in which truncated peptides could generate dominant negative and positive mutations to cause more apparent phenotypes in plants. These efforts will be helpful in the further applications of CRISPR/Cas9 genome editing to improve plant growth and stress responses.

## Methods

### Plant material, growth, and generation of transgenic *Arabidopsis* plants

*Arabidopsis thaliana* ecotype Columbia (Col-0) was used. Plant growth conditions were described previously^52^,^53^. Seeds of the *ost2* mutants (CS67805 and CS67806)^45^ were obtained from the Arabidopsis Biological Resource Center. The CRISPR/Cas9 plasmids were transformed into *Agrobacterium tumefaciens* strain GV3101 and introduced into *Arabidopsis* by the floral dipping method as described previously^35^. Transgenic plants were selected on medium containing 0.8 μg/mL blasticidin S, and by the appearance of GFP fluorescence^35^. Plant growth conditions for transgenic plants were described previously^35^.

### Construction of CRISPR/Cas9 vectors

For the construction of all-in-one CRISPR/Cas9 vectors, a plant codon-optimized (GC-rich version) *Streptococcus pyogenes Cas9*, namely “*fcoCas9*”, fused to *GFBSD2*^35^ via a self-cleaving 2A peptide was inserted between CaMV35SΩ, the Arabidopsis *elongation factor 1* (At1g07940; 2.57 kbp)^41^, or *histoneH4* (At5g59690; 0.92 kbp)^42^ promoter and the *hsp18.2* terminator^54^ in the binary vector pCAMBIA through restriction enzyme-mediated excision and ligation reactions. In these constructs, using PCR primers, a FLAG-tag and a NLS were fused to fcoCas9 at the N-terminus, and a NLS was also fused to the C-terminus of fcoCas9. For the gRNA cassettes in these vectors, the *AtU6-1* promoter and sgRNA, and the *AtU6* 3’end were used, and a custom designed gRNA can be inserted into the *Bsa*I site in between the *AtU6-1* promoter and the gRNA. All oligomers for sgRNA used in the CRISPR/CAs9 vectors are shown in Supplemental Table 1.

### gRNA design website

To design efficient gRNAs easily while avoiding “off-targets”, UI/UX of the “focas” website (http://focas.ayanel.com/) was designed and developed using the stand-alone softwares, CasOT^38^ and sgRNA Designer^39^ ordered from Ayanel & Co (Tokyo). The python script of on_target_score_calculator.py was customized to our vector constructions. Using our website, not only complete genome data but also draft genomic sequences of various plant species can be used to design suitable gRNAs without off-targets, and evaluations of “on-target” can be implemented.

### Analysis of mutations at CRISPR/Cas9 target sites

DNA was extracted from each of a T1 or T2 Arabidopsis plant that had high GFP fluorescence. To analyze mutations in the CRISPR/Cas9 transgenic plants, a 150–300 bp region surrounding the target locus of the tru-gRNAs was amplified by PCR using PrimerSTAR GXL DNA polymerase (Takara, Japan). For the Cel-1 assay, the PCR products were digested with the Surveyor Mutation Detection Kits (Transgenomic, USA) or Guide-it^TM^ Mutation Detection Kit (Takara, Japan) according to the manufacturer’s instructions. In PCR-RFLP analysis, the PCR products for *ABI4* were digested with *Bam*HI, a specific restriction enzyme whose recognition site is 4 bp upstream of the PAM. For HMA, the PCR fragments were also analyzed directly using a microchip electrophoresis system with MCE202 MultiNA (Shimadzu, Japan). To determine the sequences of CRISPR/Cas9-induced mutations in Arabidopsis, the PCR products were cloned into the pCR TOPO vector and sequenced as described previously^35^. Amplicon deep sequences were performed using the batched PCR products from 15–30 randomly selected T1 plants with high GFP fluorescence in the CRISPR/Cas9 transgenic plants. Multiplex identifiers-labelled PCR products were purified using the Wizard SV Gel and PCR Clean-Up System (Promega, USA) and sequenced on an Illumina MiSeq platform at FASMAC Co. (Kanagawa, Japan). A total of 33,855 reads for ABI4_CRISPR, 36740–79212 reads for GL1_CRISPR1, 1417-6511 reads for OST2_CRISPR1, and 1154–1570 reads for OST2_CRISPR2 were used for data analysis performed using the CLC genomics workbench (CLC bio, USA) and INTEGRATIVE GENOMICS VIEWER 2.3^53^,^55^,^56^ as previously described^26^. All primers for PCR are listed in the Supplemental Table 1.

### Quantitative RT-PCR and protein gel blot analysis

Total RNA was extracted with Trizol Reagent (Invitrogen, CA) from 3-week-old *Arabidopsis* grown on GM agar plates. Mir-X™ miRNA qRT-PCR SYBR Kit (Takara, Japan) was used for the isolation of sgRNA expressed in plant cells. Real-time quantitative PCR was performed as described previously^53^. For detection of sgRNA expression levels, a sgRNA specific primer designed at the site of tracrRNA (Supplemental Table 1) and the primer contained in the Mir-X™ miRNA qRT-PCR SYBR Kit were used. All primers used for qRT-PCR are listed in Supplemental Table 1.

For preparation of total protein extracts, 3-week-old transgenic plants were ground in liquid nitrogen and thawed in extraction buffer (25 mM Tris-HCl, pH 8.0, 5 mM MgCl_2_, 1 mM CaCl_2_, and a protease inhibitor mixture (Roche Applied Science) using a mortar and pestle. The extracts were then centrifuged at 10,000 × g for 10 min at 4 °C and the supernatant was used for the gel blot analysis. The gel blot analysis was performed as described previously^52^,^53^ using monoclonal anti-Cas9 antibody (Acrive motif, USA) and anti-GFP antibody (Santa Cruz Biotechnology, USA).

### Analyses of stomatal responses

The experimental procedures for analyses of stomatal responses were performed as described previously^53^. In ABA-induced stomatal closing assays, epidermal peels from leaves of 4-week-old plants grown at 22 °C under 16 h light/8 h dark in 50% RH were incubated for 2 h in a solution containing 10 mM KCl, 0.2 mM CaCl_2_, and 10 mM MES-KOH (pH 6.15) under white light (300 μmol m^−2 ^s^−1^). The peeled strips were subsequently incubated in the same buffer with ABA. Guard cells were photographed under a light microscope. Thirty stomatal apertures were measured for each experiment. Analysis of fresh weight loss was performed using detached rosette leaves of plants at the same developmental stage and size of 3-week-old plants. Two shoots per plant were excised and maintained in a growth chamber at 30% RH. Fresh weights were measured at the time periods indicated, and ten plants of each genotype and transgenic line were analyzed in independent experiments repeated five times.

Stomatal conductance of mature leaves of four-week-old plants grown on soil pots at 22 °C under 16 h light/8 h dark in 50% RH were detected using SC-1 Leaf Polometer (Decagon, USA). Twenty leaves were measured for stomatal conductance. Thermal images of plants were detected by using thermography apparatus with an infrared camera (Inf Rec R500, Nippon Avionivs, Japan). All experiments measuring stomatal responses were repeated three times.

## Additional Information

**How to cite this article**: Osakabe, Y. *et al.* Optimization of CRISPR/Cas9 genome editing to modify abiotic stress responses in plants. *Sci. Rep.* **6**, 26685; doi: 10.1038/srep26685 (2016).

## Supplementary Material

Supplementary Information
