# Supplementary material for: Optimization of CRISPR/Cas9 genome editing to modify abiotic stress responses in plants
Source: Sci Rep. 2016 May 26;6:26685. doi: 10.1038/srep26685 (PMC4880914; doi:10.1038/srep26685)

**Full title:**

Optimization of CRISPR/Cas9 genome editing to enhance abiotic stress responses in plants.

**Authors:**

Yuriko Osakabe^1,2^, Takahito Watanabe^2^, Shigeo S Sugano^2^, Risa Ueta^2^, Ryosuke Ishihara^2^, Kazuo Shinozaki^1^, Keishi Osakabe^2^

**Authors address:**

^1^RIKEN Center for Sustainable Resource Science, 3-1-1 Koyadai, Tsukuba, Ibaraki 305-0074, Japan

^2^Center for Collaboration among Agriculture, Industry and Commerce, The University of Tokushima, 3-18-15 Kuramoto-cho, Tokushima 770-8503, Japan


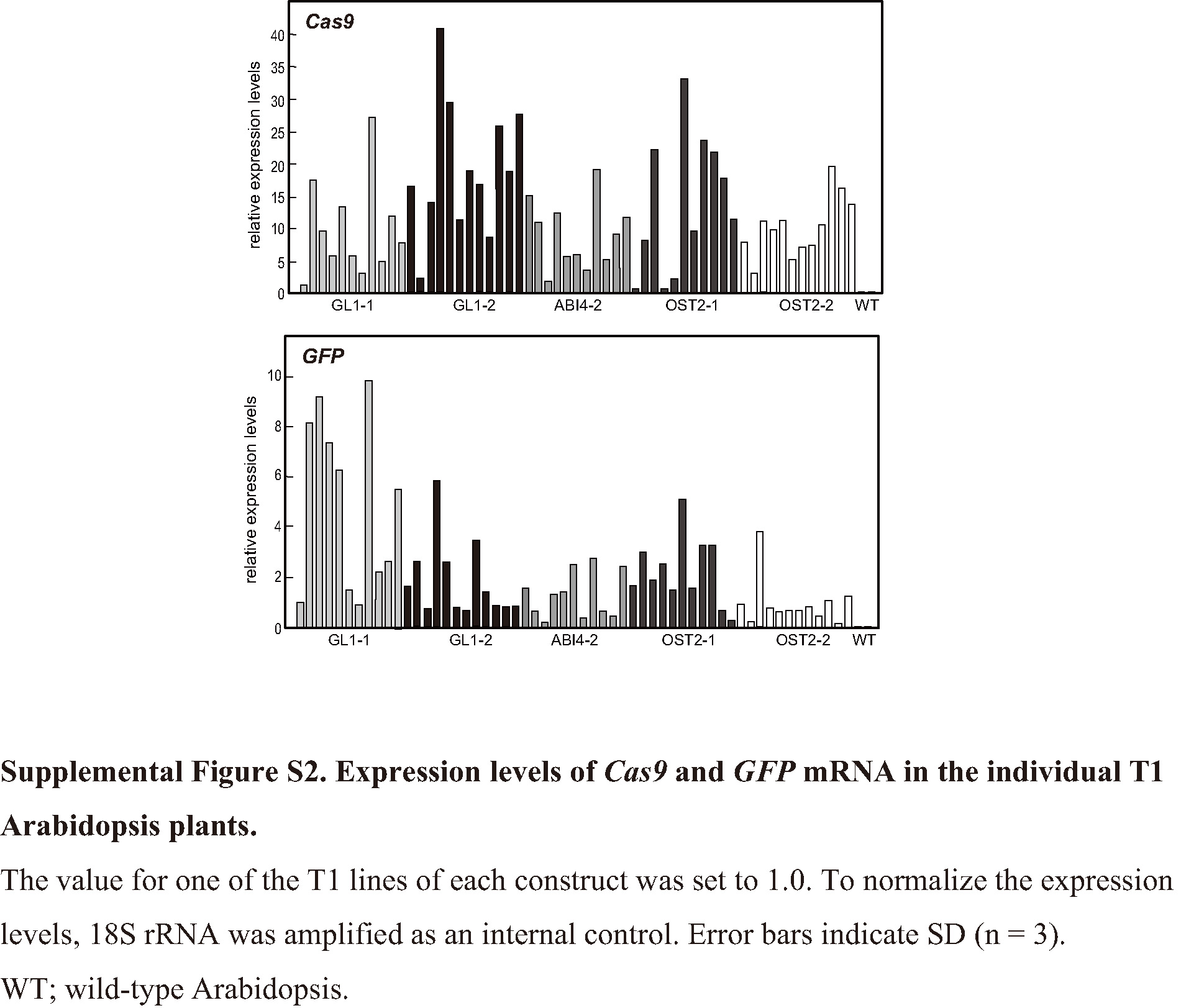


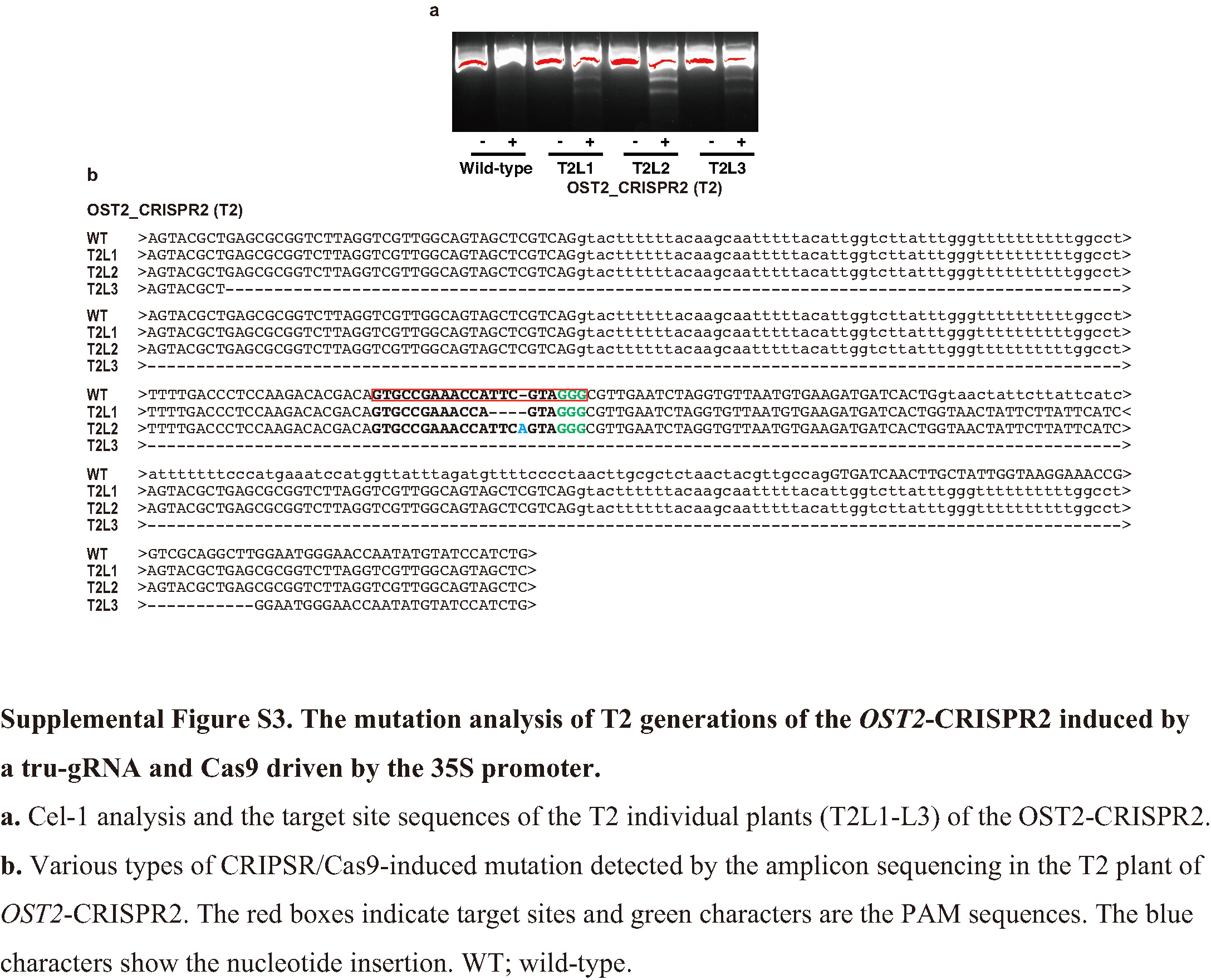

Supplement: Supplementary Information [file srep26685-s1.doc]
